# Paediatric IR: the evidence for best practice is growing

**DOI:** 10.1186/s42155-020-00201-7

**Published:** 2021-01-06

**Authors:** Alex M. Barnacle, Anne Marie Cahill

**Affiliations:** 1 Department of Radiology, Great Ormond Street Hospital for Children, London, WC1N 3JH UK; 2 Department of Radiology, Children’s Hospital of Philadelphia, 3401 Civic Center Blvd, Philadelphia, PA 19104 USA

It is immensely gratifying to see four recent papers in this journal dedicated to paediatric interventional radiology (PIR) (Patel and Cahill 2021; Gill and Shivaram 2020; Gibson and Barnacle 2020; Shaikh and Gomez Munoz 2021). They detail the latest evidence for a range of complex interventions in children, demonstrating where best practice lies in this highly specialised field. These reviews illustrate that the many of the latest advances in adult IR are applicable in children, despite the concerns of many adult IR practitioners that such interventions may be too risky or perhaps futile. Each paper, however, also highlights the frustrations common to every paediatric interventional radiologist. These include the limited evidence base available to date, the lack of paediatric-sized equipment and the challenges of developing and maintaining IR competencies with small caseloads, often focussed in only a few specialised centres.

The old adage that ‘children are not just small adults’ remains true. To practice PIR, we need to understand what is possible in small children, as these papers amply demonstrate. A few interventions may not be possible in small territories but there are usually alternatives, such as axillary access for angiography, which work extremely well in children (Roebuck et al. 2010). But most interventions can be modified for small patients, using the inventive and adaptive skills IR is known for. Even more importantly, we need to understand differences in the pathology when approaching a paediatric case, such as routinely needing 16–20 cores for neuroblastoma tumour biopsy profiling, when to stent a malacic bronchus and why adult-based imaging algorithms for gastro-intestinal bleeding are usually less helpful in children.

The paper by Patel and Cahill on renovascular hypertension in children highlights the substantial healthcare differences that a child faces compared to an adult with a similar condition (Patel and Cahill 2021). Blood pressure is inconsistently measured in children, unlike in adults, and childhood hypertension is usually an incidental diagnosis. This means that children with significant hypertension, presenting late, are at much higher risk of long-term cardiac and cerebrovascular events and renal impairment than an adult arteriopath diagnosed early on routine screening. Their paper also emphasises the subtle but very important differences in the aetiologies underlying hypertension in adults and children and why this is critical to shaping our approach to intervention. In adult IR, renal artery stenting is possible if required in the management of atherosclerotic renal artery disease but in hypertensive children with renovascular disease, the underlying pathology is usually a form of fibromuscular dysplasia and avoiding stents is key due to arterial size. Here, there should be a much stronger emphasis on angioplasty, which gives excellent results for the majority of patients over time.

Gill and Shivaram have written a detailed overview of the management of systemic venous occlusions in children (Gill and Shivaram 2020). Their paper recognises both the rising incidence of thromboembolic disease in paediatric medicine and the devastating effects of central venous occlusion in children with chronic disease who require lifelong central venous access. The authors demonstrate what is possible in small patients and advocate for proactive intervention to give children the best possible options through their adult life. It is encouraging to see some growth in evidence with regard to venous stenting, patency rates and the use of IVUS in children, but more data is needed.

The paper by Gibson and Barnacle emphasises some intriguing differences in the management of vascular anomalies in adults and children (Gibson and Barnacle 2020). Their paper gives due focus to vascular tumours which are far rarer and less consequential in adults but which can be life-threatening in the first few days of life. Life-saving embolisation is a potential option for neonates with rapidly involuting congenital haemangiomas (RICHs) presenting in advanced cardiac failure; it is beholden on all IRs to have some grasp of these pathologies so that these vulnerable patients are offered timely, safe and effective treatment when medical therapy fails. The paper also details relatively newly described pathologies such as fibroadipose vascular anomaly (FAVA) and the latest thinking around overgrowth syndromes. If we describe ourselves as a specialty that encompasses the treatment of vascular anomalies, we all should understand something about these rarer conditions and ensure we are speaking the same language.

Gomez Munoz and Shaikh provide an excellent overview of current advances in endovascular paediatric interventional oncology (IO) (Shaikh and Gomez Munoz 2021). They detail the unique challenges of calculating therapeutic dose in small bodies with proportionally very large surface areas, of effective drug delivery in extremely small blood vessels and the management of post-embolisation syndrome, which is far more common in small children than adults. They rightly state that the largely unexplored frontiers of paediatric IO present an exciting opportunity for IRs. They discuss the increasing evidence for TACE in paediatric-specific diseases such as retinoblastoma and IO’s increasing role in limb salvage surgery and palliation. Crucially, the authors call for large, multi-institutional registries to provide data from which to study the efficacy and safety of these novel therapies on a far larger scale.

These papers detail some of the small but growing evidence base for intervention in children. The specialty has come a long way since the first PIR papers were published in the early 1980s. The first PIR workforce survey in 2007 identified just 110 PIRs practising worldwide, rising to 177 in a follow-up survey 10 years later (Sidhu et al. 2007; Kaufman et al. 2017). This year, in part due to the advantages of new online conference accessibility during the COVID-19 pandemic, over 600 delegates from 22 countries registered for the Society for Pediatric Interventional Radiology (SPIR) annual congress. This groundswell of interest may be influenced by increasing visibility of the speciality through the work of publications such as this and organisations such as SPIR, but we believe it also speaks to a growing interest in a speciality that brings great rewards for those searching for greater meaning in their work. There is immense satisfaction in giving a family real hope for their child and providing care that often means accompanying a child throughout their journey to adulthood.

Harned et al. have detailed the challenges to developing PIR around the world (Harned 2nd et al. 2018). These are not dissimilar to those adult IR faced as a specialty 20 years ago. They include the growth of training programs and formal PIR curricula, wider recognition of the need for PIR services by the healthcare organisations that commission them, collaboration with industry in the development of paediatric-appropriate equipment and the need for ever more clinical data. This latter need must be addressed by the creation of more PIR registries, the inclusion of IR options in paediatric clinical trials and partnership with influential stakeholders such as the Children’s Oncology Group (COG) and the International Society of Paediatric Oncology (SIOP).

It is imperative that we strive to eliminate the inequality that means the vast majority of children even in developed countries are denied modern, minimally-invasive, safe and highly effective IR procedures that are routinely offered to adults. Significant progress has been made in recent years by a small, highly dedicated PIR workforce but there is still much more to be done.

## Data Availability

Not applicable.
